# Antiproliferative Mechanisms of Metformin in Breast Cancer: A Systematic Review of the Literature

**DOI:** 10.3390/ijms26010247

**Published:** 2024-12-30

**Authors:** Aiman Moldasheva, Assem Zhakupova, Mohamad Aljofan

**Affiliations:** 1 Department of Biomedical Sciences, School of Medicine Nazarbayev University, Astana 010000, Kazakhstan; aiman.moldasheva@nu.edu.kz (A.M.); a.zhakupova@nu.edu.kz (A.Z.); 2 Laboratory of Drug Discovery and Development, Center for Life Sciences, National Laboratory Astana, Nazarbayev University, Astana 010000, Kazakhstan

**Keywords:** metformin, breast cancer, systematic review, AMPK, mTOR

## Abstract

Metformin is an antidiabetic drug with reported potential antiproliferative activity against different cancer types including breast cancer. However, the mechanism of action of how metformin can induce its antiproliferative activity is still unclear. Thus, the current study is a systematic review of the literature aiming to explore the reported antiproliferative mechanisms of metformin against breast cancer. The study included seventeen research articles that describe different mechanisms of action against breast cancer. While the majority of the studies confirm the antiproliferative potential of metformin, albeit at different potencies, there appear to be various mechanisms and factors that can influence this effect. There are a number of questions yet to be answered pertaining the use of metformin as an anti-cancer agent, warranting further investigation into this emerging area of research.

## 1. Introduction

Metformin is an oral antidiabetic drug used as the first-line therapy for type 2 diabetes mellitus (T2DM) and has been on the market for more than 70 years [1,2]. It has several off-label uses including for polycystic ovarian syndrome (POS) [3,4], weight control [5,6] and anti-aging [7,8]. Recently, metformin was reported to inhibit cellular proliferation of cancer cells, including oral [9], breast [1,2,10,11], pancreatic [10,12,13,14], prostate [15], colorectal [16], lung [17], gastrointestinal [18], thyroid [19], and ovarian [20] cells, as well as suppressing angiogenesis [21,22]. The most likely antiproliferative mechanism of metformin was reportedly associated with its ability to activate AMPK and LKB1, which inhibits the mechanistic target of rapamycin (mTOR) (a commonly dysregulated pathway in cancer) and consequently inhibit protein synthesis and cell proliferation [23]. Others suggested that the observed antiproliferative potential is due to the drug’s ability to lower blood glucose levels, and increase insulin sensitivity [24]. However, the exact antiproliferative mechanism of metformin is not completely understood. Thus, the current manuscript is a systematic review of the literature aiming to analyze and characterize the reported antiproliferative mechanism of metformin, with a particular focus on breast cancer. Indeed, exploring the potential mechanism or mechanisms of how metformin induces its antiproliferative effect against breast cancer might result in improving the treatment options for the otherwise fatal disease.

## 2. Results

The database search yielded a total of 422 articles, of which 380 articles were removed (77 review articles, 53 clinical trials, and 250 other articles with non-related topics). A further 25 papers were removed in the second round of screening for irrelevancy and duplications.

**Figure 1:**
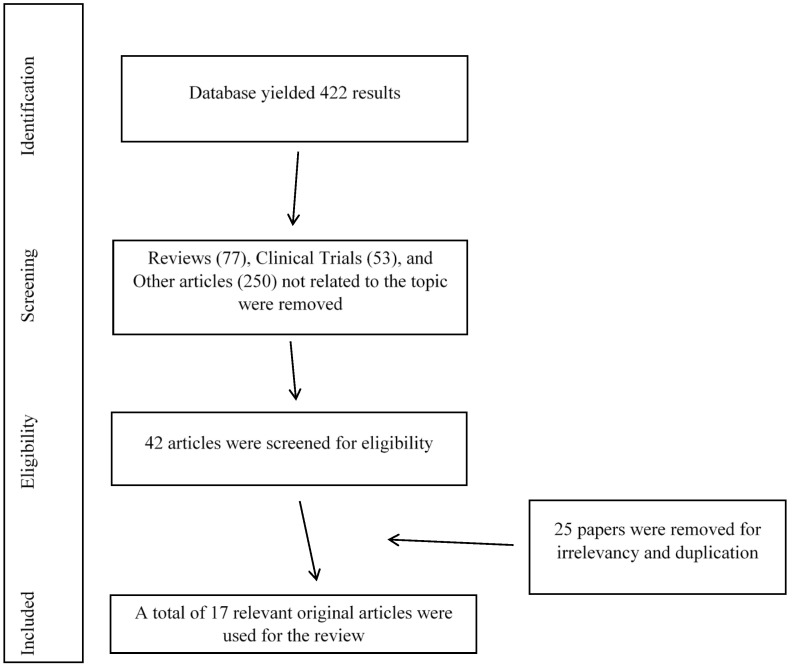
The flow of information search strategy. Initial search resulted in 422 articles. After screening and abstract reviewing, 17 original articles were chosen to be covered in the systematic review.

Therefore, a total of 17 study articles were included in the present systematic review. The flow of information searched through the different phases of the current review is shown in Figure 1, and the list of included articles with a summary of their findings is shown in Table 1.

### 2.1. Metformin Induces Apoptosis

Several studies reported that metformin treatment induces apoptosis and cell cycle arrest in breast cancer cell lines [25,26,27,28,29]. The results of all studies agree that metformin treatment increases the Bax/BCL-2 ratio, which is a cellular switch for apoptosis initiation. Moreover, Queiroz et al. and Sharma et al. demonstrated that metformin treatment increases the levels of active executioner caspase 3 and caspase 7, which carry out the ultimate cleavage of the cellular proteins [25,26].

#### 2.1.1. Metformin Affects the AMPK-FOXO3a-CyclinD1/p27 Signaling Pathway

In order to elucidate which signaling pathway leading to apoptosis is altered by metformin, Queiroz et al. evaluated the expression of different mRNAs and proteins in the MCF-7 cell line. The authors found that metformin increased the phosphorylation of AMPK (Thr172) and reduced the phosphorylation of p70S6K (Thr389), suggesting that metformin leads to the activation of AMPK and inhibition of mTOR. This is in accordance with previous reports, where metformin has been shown to work through the AMPK/mTOR signaling pathway [42,43]. Moreover, metformin was shown to inactivate Akt (protein kinase B), which is an important player in the PI3K/Akt/mTOR pro-survival signaling pathway.

Interestingly, metformin was also shown to increase the expression of pro-apoptotic transcription factor FOXO3a, which was previously reported to be negatively regulated by Akt [44]. Queiroz et al. showed that, in addition to Akt, FOXO3a expression can also be up-regulated by the activation of AMPK and accumulation of ROS. Metformin was reported to affect both. Therefore, metformin-induced down-regulation of Akt, activation of AMPK, and the accumulation of ROS all contribute to the activation of FOXO3a. The importance of FOXO3a in cancer development and treatment has been recently recognized, as it was found to regulate important cellular processes such as cell proliferation, DNA damage response, cell cycle arrest, and apoptosis [45,46]. FOXO3a also plays an important role in dealing with oxidative stress [47]. Overexpression of FOXO3a inhibited cellular proliferation and tumorigenesis, and, hence, FOXO3a seems to function as a tumor suppressor [48].

**Figure 2:**
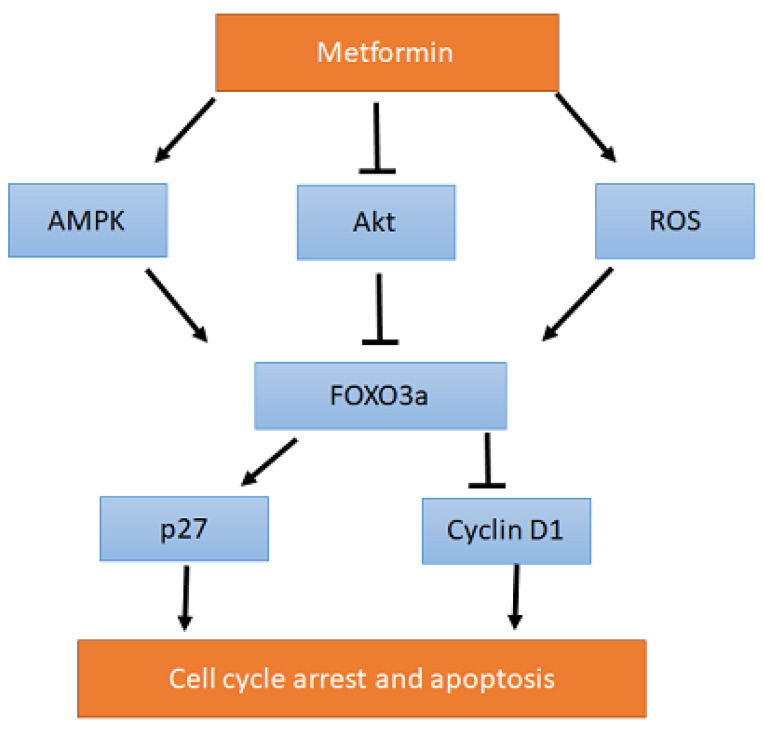
Possible pathways of antiproliferative mechanisms of metformin. Metformin induces overexpression of FOXO3a through up-regulation of AMPK, ROS, and the inhibition of Akt. FOXO3a, in turn, increases expression of cell cycle inhibitor p27 and decreases expression of Cyclin D1, which can ultimately result in cell cycle arrest and apoptosis.

Downstream of FOXO3a, metformin increased the expression of cell cycle inhibitor p27 and decreased the expression of Cyclin D1, both of which support the hypothesis that metformin induces cell cycle arrest and apoptosis (Figure 2).

#### 2.1.2. Metformin Induces ROS-Mediated Apoptosis

Excess accumulation of ROS can damage cellular macromolecules such as DNA, RNA, and proteins, which can ultimately lead to cell cycle arrest and cell death. Cancer cells have developed mechanisms to deal with excess ROS by up-regulating the expression and function of antioxidant defense systems. Therefore, the initiation of oxidative stress via ROS accumulation is a potential mechanism for initiating apoptosis in cancer cells.

Several authors report that metformin induces the accumulation of ROS, which can be responsible for the subsequent initiation of apoptosis [25,29,30]. Results from Queiroz et al.’s study demonstrated that metformin exposure resulted in a significant increase in ROS after 72 h treatment in MCF-7 cells. Metformin also increased H_2_O_2_ production, which could be reversed by concomitant exposure to catalase. The involvement of oxidative stress in the cytotoxic effect of metformin was further supported by partial reversal of the metformin-induced cell death by antioxidant enzymes: superoxide dismutase, catalase, and apocynin.

In another study by Huynh et al., metformin was shown to induce proline dehydrogenase/proline oxidase (PRODH/POX)-dependent apoptosis via accumulation of ROS [30]. PRODH/POX is an enzyme that catalyzes the conversion of proline into Δ1-pyrroline-5-carboxylate (P5C) with concomitant production of ROS, which can initiate intrinsic and extrinsic apoptosis. Additionally, PRODH/POX is known to be activated by AMPK ligands.

Metformin was shown to be cytotoxic to both MCF-7WT (wild type) and MCF-7crPOX (PRODH/POX knock-out) cells, but to a lesser extent in the latter, suggesting that PRODH/POX can play a role in the mechanism of action of metformin. Moreover, metformin was shown to be more effective in producing ROS and inducing apoptosis in MCF-7WT compared to MCF-7crPOX.

Interestingly, metformin did not change the expression of PRODH/POX but increased the concentration of its substrate, proline, by inhibiting collagen biosynthesis and increasing prolidase expression and its activity. Proline is one of the major amino acids that make up collagen; hence, decreased collagen synthesis spares proline. Prolidase is the enzyme responsible for the recovery of proline from degradation products of collagen; activation of which also leads to increased proline concentrations. Authors believe that a metformin-induced increase in proline concentration leads to its increased conversion into P5C with concomitant production of ROS, which can at least partially contribute to metformin-induced apoptosis.

Another piece of evidence supporting the hypothesis of ROS-mediated apoptosis comes from a study by Gao et al. [29]. Authors showed that metformin in a dose- and time-dependent manner decreased cell viability and colony formation in MDA-MB-231 and MDA-MB-435 cell lines. Additionally, metformin was shown to decrease mitochondrial membrane potential and ATP production and to increase ROS. Metformin exposure also resulted in increased apoptosis, which was further confirmed by increased expression of pro-apoptotic Bax and decreased expression of anti-apoptotic BCL-2 and MCL-1. Overall, the authors believe that metformin-induced ROS accumulation leads to the induction of mitochondria-mediated intrinsic apoptosis.

#### 2.1.3. Metformin Induces mTOR Protein Degradation

As previously mentioned, metformin has been repeatedly reported to alter the AMPK/mTOR signaling pathway. Alalem et al. demonstrated that breast cancer cells, compared to noncancerous cells, not only have higher total protein levels of mTOR but also exhibit higher stability for this protein [31]. Metformin, in turn, was demonstrated to decrease the total mTOR protein level in MCF-7, which correlated with its inhibitory effect on cell proliferation and migration. This has led to the hypothesis that metformin increases mTOR protein degradation.

Interestingly, authors found that metformin neither induces vacuolization of cytoplasm (characteristic of autophagy) nor does it increase mTOR proteasomal degradation. Metformin, instead, induced the accumulation of mTOR protein in a perinuclear aggresome, which is an accumulation of misfolded proteins for later clearance by autophagy [49].

### 2.2. Metformin Induces Ferroptosis

Ferroptosis is a recently discovered form of cell death distinct from apoptosis, which is caused by an iron-dependent accumulation of lipid peroxidation products [50]. Biochemically, ferroptosis can be characterized by the accumulation of Fe^2+^, ROS, and the depletion of glutathione and inactivation of glutathione peroxidase 4 (GPX4) [51]. Morphologically, ferroptosis manifests itself as a loss of cytoplasmic integrity, oncosis, smaller sizes of mitochondria, shrinkage of mitochondrial cristae, and an increase in membrane density [52].

Ferroptosis occurs as a result of a disbalance between pro- and antioxidant cellular mechanisms. Cancer cells tend to accumulate Fe^2+^, which can lead to the production of ROS through Fenton reactions [53,54]. Increased oxidative burden, in turn, can lead to an increase in lipid peroxidation and induction of ferroptosis as a result. This becomes especially critical when cellular antioxidant mechanisms are compromised. As such, glutathione and GPX4, have been shown to play important roles in ferroptosis. Glutathione is an antioxidant synthesized mainly from cysteine. Cysteine availability, in turn, relies on the cystine/glutamate transporter system (Xc-, a heterodimer consisting of SLC7A11 and SLC3A2 subunits) [55]. Hence, down-regulation or inhibition of this transporter system ultimately leads to a decreased production of glutathione and can overweigh the balance in favor of ferroptosis. Additionally, SLC7A11 was reported to be up-regulated in several cancers [56]. GPX4 is an enzyme that catalyzes a reduction in peroxides utilizing the glutathione (GSH), hence serving as an antioxidant. GPX4 is believed to be one of the key regulators of ferroptosis.

Ferroptosis has recently gained a lot of attention in cancer treatment research because it was shown that tumor cells that can evade other forms of death are susceptible to ferroptosis [57]. Therefore, targeting ferroptosis has turned out to have attractive potential for cancer treatment. Interestingly, several studies have shown that metformin can induce ferroptosis in breast cancer cell lines [32,33]. Hou et al. demonstrated that metformin induces ferroptosis in the MDA-MB-231 cell line, which was shown by the accumulation of ROS, Fe^2+^, and decreased expression of GPX4, similar to the effects of known ferroptosis inducers, RSL3 and erastin [32]. Moreover, metformin up-regulated the expression of miR-324-3p, the overexpression of which resulted in decreased cell viability and decreased expression of GPX4. Since GPX4 is one of the main defense mechanisms against lipid peroxidation and is the principal regulator of ferroptosis, authors believe that metformin induces ferroptosis in breast cancer cells through the regulation of the miR-324-3p—GPX4 signaling pathway. This was further supported by in vivo studies, where metformin induced ferroptosis by up-regulation of miR-324-3p in a breast cancer xenograft mice model.

Similarly to the study of Hou et al., Yang et al. demonstrated that metformin exposure leads to ferroptosis in breast cancer cell lines MCF-7 and T47D [33]. However, according to Yang et al., metformin induces ferroptosis through decreased UFMylation of SLC7A11 protein, leading to its decreased stability. UFMylation is one form of post-translational modifications that involves the attachment of Ubiquitin Fold Modifier-1 (UFM-1) to target proteins. This was shown by a metformin-induced decrease in the expression of SLC7A11, UFM1, and decreased levels of UFMylated SLC7A11. As mentioned above SLC7A11 participates in transporting the cystine required for GSH synthesis; therefore, decreasing the stability of SLC7A11 by using metformin can lead to a depletion of the GSH pool and ultimately to the induction of ferroptosis. The importance of both SLC7A11 and UFM1 for ferroptosis was shown by the down-regulation of both proteins by a known ferroptosis inducer—erastin. The authors of this study also showed that metformin induces ferroptosis by regulation of the UFM1/SLC7A11 pathway in an AMPK-independent manner.

Overall, there is agreement that metformin can induce ferroptosis in breast cancer cells. Mechanisms of ferroptosis induction, however, are different according to different studies, but they are not opposing. On the contrary, metformin was shown to induce ferroptosis by affecting two of the most prominent regulators of ferroptosis: GPX4 and SLC7A11.

### 2.3. Metformin Inhibits Epithelial-to-Mesenchymal Transition (EMT)

EMT is a phenotypic transition of epithelial cells into mesenchymal that is crucial for normal embryonic development. In adults, however, EMT is mostly associated with pathological processes contributing to cancer initiation, development, and progression [58]. EMT is characterized by the loss of epithelial characteristics such as intercellular attachments, apicobasal polarity, disruption of tight junctions and acquisition of mesenchymal characteristics such as a spindle-like morphology, increased motility, protease-dependent invasion, stem cell traits, and therapy resistance [59]. EMT plays an important role in the metastatic potential of cancer cells, chemotherapy resistance, and cancer stem cell generation. Hence, EMT has become an attractive target for cancer therapy, especially for patients with advanced cancer.

According to Sharma et al., metformin inhibited cell migration in the MDA-MB-231 cell line, which was likely due to the prevention of EMT [26]. This was demonstrated by increased expression of epithelial markers such as E-cadherin and keratin 19 and decreased expression of mesenchymal markers such as vimentin, N-cadherin, Zeb1, and Zeb2.

Similarly, Huang et al. demonstrated that metformin inhibited cellular migration in MCF-7 cells in a dose-dependent manner (5–20 mM) [60]. This was supported by decreased expression of matrix metalloproteinases MMP2 and MMP9, which play a role in tumor cell invasiveness and metastasis. Moreover, metformin seems to negatively affect the Wnt/β-catenin signaling pathway, which is also associated with cell cycle regulation, migration, and metastasis. This was shown by metformin (20 mM)-induced down-regulation of Wnta3a, Wnt5a, and β-catenin as well as down-regulation of c-Myc, which is a target gene of Wnt/β-catenin pathway [60]. This study, however, did not find a significant difference in the expression of EMT markers E-cadherin and Vimentin between metformin-treated and control groups that were affected by metformin exposure in the study by Sharma et al.

Esparza-López et al. found that primary breast cancer cells with mesenchymal phenotype were more resistant to metformin treatment than breast cancer cells with epithelial phenotype, supporting the theory that EMT confers therapy resistance [34]. The authors also evaluated how metformin can affect mesenchymal markers in primary breast cancer cells with a mesenchymal phenotype. They found that metformin down-regulates mesenchymal markers such as Vimentin and SNAIL in a time-dependent manner [34]. The authors also demonstrated that pro-inflammatory cytokine IL-6 can induce epithelial-to-mesenchymal transition (EMT) of primary epithelial breast cancer cells. Metformin, in turn, was able to reverse EMT in IL-6-induced model by increasing the re-expression of E-cadherin and inhibiting IL-6-induced expression of Vimentin and SNAIL. Metformin’s ability to reverse IL-6-induced EMT correlated with its inhibitory effect on cell proliferation and migration.

Overall, several studies have shown that metformin can inhibit cellular migration through the inhibition of EMT. Metformin was shown to up-regulate epithelial markers such as E-cadherin and keratin-19 and down-regulate mesenchymal markers such as Vimentin, SNAIL, N-cadherin, Zeb1, and Zeb2. Moreover, metformin also inhibited the expression of metalloproteinases, which are not considered to be classical EMT markers but are known to facilitate the epithelial-to-mesenchymal transition [61].

### 2.4. Metformin Inhibits Cancer Stem Cells

Interestingly, EMT is not only associated with increased invasiveness and cellular migration but also with the ability to induce stem cell-like phenotypes in invading cancer cells [62]. Therefore, metformin’s ability to inhibit EMT can also indirectly affect cancer stem cells. In addition to indirectly affecting stem cells through the inhibition of EMT, metformin was reported by several studies to directly affect breast cancer stem cells by targeting stemness markers and stem cell-related transcription factors.

The study by Sharma et al. demonstrated that metformin treatment was able to reduce the number of colonies formed compared to control [26]. Moreover, expression of stemness markers such as BMI1 and CD44 was reduced in metformin-treated cells, suggesting that metformin can also target cancer stem cells [26].

In another study by Shi et al., metformin was shown to significantly decrease the percentage of triple negative breast cancer cells (TNBC), stem cells in HCC1806, and HCC1937 cell lines in a dose-dependent manner (20–50 mM). Metformin was found to down-regulate the expression of KLF5, which is a stem cell transcription factor, and its downstream target genes FGF-BP1 and Nanog. Nanog is a transcription factor involved in embryonic stem cell proliferation and renewal [35].

A more detailed analysis showed that metformin increases GSK3β-mediated phosphorylation of KLF5, targeting it for proteasomal degradation. An increase in GSK3β-mediated phosphorylation, in turn, happens due to metformin-induced inhibition of protein kinase A (PKA) activity [35].

In summary, authors found that metformin suppresses TNBC stem cells via the PKA- GSK3β-KLF5 pathway. The authors also demonstrated that TNBC samples have an overactive PKA- GSK3β-KLF5 pathway compared to non-TNBC cell lines. Therefore, metformin can be potentially useful for patients with TNBC, who usually have a poor prognosis.

Further support that metformin can target breast cancer stem cells (BCSCs) came from a study of Tan et al., where metformin treatment of MCF-7.SC and MDA-MB-231.SC resulted in increased expression of miR-708 and decreased expression of CD47 mRNA in both cell lines. MiR-708 is a microRNA that is known to function as a tumor suppressor and is dysregulated in several types of cancer. Tan et al. demonstrated that miR-708 expression is significantly down-regulated in BCSCs. Moreover, miR-708 overexpression in both MCF-7 and MDA-MB-231 resulted in decreased self-renewal capacity, which was demonstrated by the decreased number of generated mammospheres compared to the negative control. In addition to that, overexpression of miR-708 significantly reduced the CD44+/CD24- population, further supporting the hypothesis that miR-708 overexpression can limit the self-renewal capacity of BCSCs [36].

Cluster differentiation 47 (CD47), which was down-regulated by metformin, is a cell-surface glycoprotein, the overexpression of which in cancers is correlated with poor prognosis. Interestingly, CD47 was found to be a predicted target gene of miR-708. Tan et al. demonstrated that miR-708 overexpression in BCSCs resulted in the down-regulation of CD47 on both mRNA and protein levels [36]. Decreasing expression of CD47 by transfecting cells with shRNA resulted in a decreased number of mammospheres being formed, together with a reduction in the CD44+/CD24- population, supporting the importance of CD47 in the regulation of self-renewal capacity of stem cells. In summary, Shi et al., demonstrated that metformin can target breast cancer stem cells through regulation of the miR-708-CD47 signaling pathway [35].

### 2.5. Metformin Changes the Expression of Different miRNAs

MicroRNAs (miRNA) are small, non-coding RNAs that play an important role in the regulation of gene expression at the post-transcriptional level [63]. Recently, miRNAs have gained increased attention in cancer research because they were found to be dysregulated in cancer [64]. Interestingly, miRNAs can affect all cancer-related processes, such as proliferation, apoptosis, angiogenesis and metastasis [65]. One miRNA can modulate the expression of several genes and can act both as a tumor suppressor and as an oncogene depending on the target genes [63].

Metformin was shown to change the expression of several different miRNAs. According to Cabello et al., metformin significantly increased the expression of miR-26a in the MDA-MB-231 cell line, which correlated with its negative effect on cell viability [32]. This was further proven by the reversal of metformin-induced cell death by concomitant treatment with miR-26a inhibitor. Previously, miR-26a was reported to function as a tumor suppressor by several studies [66,67,68]. Moreover, Cabello et al. demonstrated that the overexpression of miR-26a resulted in decreased cell viability in all tested breast cancer cell lines (MDA-MB-231, MDA-MB-468, and MCF-7).

Additionally, metformin was shown to reduce the expression of miR-26a’s downstream target genes, PTEN and BCL-2. PTEN is a well-recognized tumor suppressor, and its down-regulation by metformin could not explain the anti-cancer effect of metformin. However, another miR-26a target—EZH2, which is an oncogene—was found to be down-regulated by metformin treatment in the MDA-MB-231 cell line [29]. Therefore, EZH2 could be at least one of the miR-26a downstream targets mediating the cytotoxic effect of metformin.

In a study by Cheng et al., metformin was reported to significantly up-regulate the expression of another miRNA—miR-483-3p—in primary breast cancer cells [38]. Downstream of miR-483-3p, there is a METTL3 methyltransferase, which is responsible for the installment of the most common mRNA modification—N6-methyladenosine (m6A). M6A has recently gained a lot of attention in cancer research due to its potential ability to activate oncogenes and inhibit tumor suppressors [69].

Cheng et al. demonstrated that breast cancer tissue, compared to adjacent normal tissue, has higher expression of both global m6A and METTL3 [38]. Metformin, in turn, was demonstrated to decrease m6A level and to reduce expression of METTL3 on both mRNA and protein levels, which correlated with a metformin-induced decrease in proliferation and colony formation. Furthermore, metformin was found to up-regulate the cell cycle inhibitor p21 expression via METTL3-associated m6A modification, which was further supported by the reduced amount of m6A modified p21 mRNA in the METTL3 knockdown experiment [38].

MiR-483-3p’s ability to regulate METTL3 was demonstrated by abolishing the effect of metformin on METTL3 down-regulation and the inhibition of cell proliferation upon treatment with an the miR-483-3p inhibitor. The authors conclude that the anti-cancer effects of metformin can be attributed to its effect on the regulation of the miR-483-3p/METTL3/m6A/p21 pathway.

### 2.6. Metformin Decreases Cholesterol Level

Cholesterol is an important structural component of cellular membranes, and rapidly dividing tumor cells have increased demand for cholesterol. Moreover, cholesterol is a precursor of steroid hormones, which are also known to play a role in breast cancer development [70]. Therefore, the depletion of cholesterol can be a potential mechanism for breast cancer treatment.

Interestingly, patients taking metformin to control Type II diabetes have been shown to have lower levels of LDL cholesterol [71]. Sharma et al. evaluated the effect of metformin on cholesterol levels and how this could contribute to the antiproliferative effect of metformin [26]. Notably, treatment of MDA-MB-231 cells with metformin resulted in a dose-dependent decrease in cellular cholesterol levels.

Moreover, metformin changed the expression of cholesterol-related genes in favor of cholesterol reduction: a decrease in SREBP1 (sterol regulatory element-binding protein 1), decrease in LDL receptors, and an increase in ABCA1 (ATP-binding cassette transporter 1). On the protein level, metformin exposure resulted in the down-regulation of HMGCoR (HMG-CoA reductase) and SREBP1. HMGCoR is a rate-limiting enzyme in the biosynthesis of cholesterol; hence, decreased levels of HMGCoR will result in decreased synthesis of cholesterol. SREBP1, in turn, plays an important role in the initiation of lipogenesis in the liver. A decrease in SREBP1 will lead to decreased synthesis and storage of fat.

Overall, these data suggest that metformin can decrease cellular cholesterol levels by modulating cholesterol-regulating proteins such as HMGCoR, LDL receptors and SREBP1. Interestingly, simultaneous treatment of cells with metformin and cholesterol reversed the anti-cancer effects of metformin such as decreased cell viability, decreased cell migration, decreased colony formation and reversed expression of cancer-associated genes. This further supports the hypothesis that the anti-cancer effects of metformin, at least partially, are mediated by its effect on cellular cholesterol levels.

### 2.7. Metformin Inhibits Angiogenesis Through the Down-Regulation of PDGFβ

Wang et al. demonstrated that metformin treatment leads to the down-regulation of PDGFβ in 4T1 breast cancer cells, which correlated with its positive effect on vessel functionality [39]. Metformin was shown to improve vascular functionality by increasing Lectin+ vessels, decreasing tumor hypoxia and reducing vessels’ leakiness. Moreover, metformin was shown to decrease the compression of cancer cells and to reduce microvessel density. The dependence of metformin on PDGFβ signaling was further supported by the abrogation of metformin-induced vascular effects by blockade of PDGFβR.

Overall, by down-regulating the PDGF-β, metformin seems to inhibit excessive angiogenesis characteristics of metastatic tumors and improve vascular functionality, which, in turn, results in improved delivery of other chemotherapeutic drugs.

### 2.8. Metformin Can Overcome Drug Resistance by Modulating the SCRIB-YAP Signaling Pathway

Yes-associated protein (YAP) is a protein that is commonly associated with chemotherapy resistance. YAP is a transcriptional co-activator, which, upon translocation to the nucleus, can control the expression of genes involved in cell proliferation, migration, and apoptosis [72]. YAP is also one of the components in the Hippo signaling pathway, which is known to regulate organ size, stem cell renewal, cell proliferation, and tumorigenesis [73]. Liu et al. demonstrated higher expression of YAP in drug-resistant breast cancer cell lines LCC2 (tamoxifen-resistant) and MCF7/TAX (paclitaxel-resistant), compared to drug-sensitive MCF-7, cell lines [40].

In the same study, metformin was reported to increase the phosphorylation of YAP by up-regulating the expression of SCRIB. SCRIB is a cell polarity protein, which plays an important role in maintaining apico-basal polarity of the cells. SCRIB was reported to be mislocalized or down-regulated in various cancers [74,75]. Metformin not only increases the expression of SCRIB but also increases its interaction with MST1/2 and LATS1, which ultimately leads to the inhibition of YAP nuclear localization and its transcriptional activity. Negative regulation of YAP by metformin resulted in decreased survival and invasiveness of drug-resistant cell lines.

In the drug-sensitive cell line MCF-7, however, metformin exposure resulted in the activation of the HIPPO pathway by increasing the expression of its upstream regulators KIBRA and FRMD6, which, similarly to drug-resistant cells, increased YAP phosphorylation and prevented its translocation to the nucleus and its transcriptional activity. Therefore, the overall metformin effect in drug-sensitive and drug-resistant cells is similar but the mechanisms leading to YAP-phosphorylation are different.

### 2.9. Anti-Cancer Effect of Metformin Depnds on Glucose and Other Nutrients Availability

Several studies have shown that the antiproliferative effects of metformin depend on glucose and other nutrients’ concentrations. For example, Silvestri et al. demonstrated that metformin-induced cell death in breast cancer cell lines (MCF-7, SKBR3, and MDA-MB-231) was significantly mitigated in high-glucose conditions (12.5 mM and higher); however, this did not affect the metformin-induced down-regulation of mTOR [27].

The authors also examined the effects of other nutrients on the cytotoxic activity of metformin. They found that the addition of nutrients, such as those commonly found in DMEM media, abolished the metformin effect of cell death. This suggests that the antiproliferative activity of metformin is glucose and/or nutrient dependent. This finding is in line with the recently published study by Nurzhan et al., which reported that glucose concentration could influence both the antiproliferative potency and mechanism of metformin [76].

To further confirm this, the effect of metformin on the expression of Pyruvate kinase M2 (PKM2) was evaluated. PKM2 is a kinase responsible for the last step in the glycolytic pathway of ATP production. PKM2 plays an important role in cancer because it not only affects the metabolic state of cancer cells but can also regulate the expression of genes related to cell growth and survival [77]. Metformin was found to down-regulate expression of PKM2 protein in nutrient-poor conditions, whereas expression of PKM2 mRNA was down-regulated independently of nutrient status. This indicates that metformin not only affects the expression of PKM2 mRNA, but can also affect the post-translational regulation of this kinase.

In accordance with Silvestri et al., Varghese et al. also showed that metformin works better at inhibiting cellular proliferation at low or normal glycemic conditions [41]. According to this study, metformin inhibited cell proliferation under normoglycemic (5.5 mM glucose) conditions in both tested TNBC cell lines, MDA-MB-231 and MDA-MB-468. With high-glucose (25 mM) conditions, however, metformin inhibited cell proliferation in the MDA-MB-468 cell line but not in MDA-MB-231.

Apoptosis was increased by metformin treatment in MDA-MB-468 cells at all tested glucose conditions (25 mM, 5.5 mM and zero glucose). In MDA-MB-231, however, metformin treatment reduced apoptosis in high-glucose conditions and increased apoptosis in normal glucose conditions. The potential effect of metformin on mTOR signaling pathway was evaluated by measuring mTOR signaling pathway-associated proteins: pmTOR (S2448), p4EBP1 (T37/46), pS6 (S235/236), and ps6 (S240/244). Metformin was shown to down-regulate the expression of all tested mTOR-associated proteins in glucose-zero conditions compared to normal and high glucose concentrations, suggesting that metformin works more effectively in inhibiting the mTOR pathway in the glucose-starved conditions.

The anti-cancer effects of metformin obviously depend on glucose and nutrient availability. According to the studies, metformin seems to work more effectively in reducing cellular proliferation and inducing apoptosis at low or normal glucose conditions as well as low nutrient concentrations. Notably, nutritional status affected metformin response differently in different breast cancer cell lines. Metformin induced apoptosis in MDA-MB-468 regardless of nutritional status, which was not the case in MDA-MB-231. Therefore, the anti-cancer effects of metformin depend not only on the nutritional status but also on the genotype of the cell line.

## 3. Discussion

Metformin is a safe, well-tolerated drug with a long history of usage as an antidiabetic agent, and recently has been investigated for various other biological effects, including anti-cancer potential. This systematic review of the literature analyzed the reported anti-cancer activity of metformin in breast cancer from a mechanism of action perspective. Analysis of the available data confirmed that metformin has an antiproliferative effect in breast cancer in vitro and in some cases in vivo.

However, the antiproliferative mechanisms of metformin remain undetermined, with several studies having suggested that metformin achieves its antiproliferative mechanism in breast cancer cells via inducing cellular apoptosis by regulating the AMPK/mTOR signaling pathway [23,78]. For example, Deng et al., reported that metformin induces apoptosis in triple-negative breast cancer cells via targeting of the Stat3 pathway, leading to cell growth inhibition [79]. Similarly, Queiroz et al. claimed that cellular treatment with metformin might increase the expression of FOXO3a, a tumor suppressor, via downstream signaling of AMPK/mTOR [25]. Metformin-induced overexpression of FOXO3a also resulted in increased expression of cell cycle inhibitor p27 and decreased expression of CyclinD1 [80]. Therefore, it is safe to assume that metformin can possibly induce cell cycle arrest and apoptosis through the regulation of the AMPK-FOXO3a-Cyclind1/p27 signaling pathway.

Another possible pathway by which metformin may induce cellular apoptosis is via ROS production, a hypothesis tested and reported by several studies; however, the mechanisms of how exactly metformin increases oxidative stress are still not fully understood. One possible mechanism described by Huynh et al. is the up-regulation of PRODH/POX-dependent ROS formation [30]. This finding is in agreement with that of Oscilowska and colleagues who reported the involvement of PRODH/POX in metformin-induced cellular apoptosis [81]. The study claimed that the activation of AMPK and PRODH/POX, increase the concentration of proline in the cytoplasm, hindering the biosynthesis of collagen, as well as stimulates PRODH/POX-dependent ROS production, thus leading to cellular apoptosis.

While metformin’s ability to induce apoptosis has been known for quite some time, its ability to induce ferroptosis, which is another form of cell death, is a recent discovery. The significance of ferroptosis induction by metformin lies in the fact that cancer cells that have been shown to escape other forms of death are susceptible to ferroptosis, an iron-dependent form of regulated cell death [82]. According to the studies by Hou et al. and Yang et al., metformin induces ferroptosis in breast cancer cells by targeting two important regulators of ferroptosis: GXP4 and SLC7A11 [32,33]. A decrease in the protein expression level of GPX4 is believed to sensitize cells to ferroptosis [82].

Interestingly, metformin was found to inhibit EMT, which is a significant contributor to cellular migration, metastasis, and stem cell-like phenotype. Metformin was shown to up-regulate epithelial markers such as E-cadherin and keratin-19 and down-regulate mesenchymal markers such as Vimentin, SNAIL, N-cadherin, Zeb1 and Zeb2. For instance, the inhibition effect of metformin on vimentin was found to be comparable to that of vimentin-siRNA suppression [83]. Moreover, metformin also inhibited the expression of metalloproteinases, which are also known to facilitate the epithelial-to-mesenchymal transition [61].

One of the interesting antiproliferative mechanisms of metformin is the metformin-induced decrease in cellular cholesterol levels and cholesterol-regulating genes (SREBP1, HMGCoR, LDL receptors), which correlated with metformin’s ability to inhibit cellular growth [26]. Since cancer cells have a high turnover rate, they heavily rely on a sufficient supply of cholesterol for growth and division. In addition, cholesterol is a precursor of steroid hormones, and breast cancer is a hormone-dependent type of cancer. Therefore, decreasing cholesterol levels can also interfere with hormone synthesis. Interestingly, statins, which are cholesterol-lowering agents, are also known to have anti-cancer activity [84]. This further supports the idea that cancer cells have an increased demand for cholesterol for cellular proliferation.

## 4. Materials and Methods

The present systematic review was performed following the guidelines of The PRISMA Statement for Reporting Systematic Reviews and Meta-Analyses of Studies That Evaluate Health Care Interventions: Explanation and Elaboration by Liberati et al., 2009 [85].

### 4.1. Search Strategy

Articles published since the first report of the anti-cancer potential of metformin in 2005 until January 13, 2023, were searched through the available databases, including the following: Medline via PubMed and EMBASE via Elsevier. We used “Metformin and Breast Cancer” as the search terms. The search was restricted to English language studies including journal articles, theses, and conference proceedings.

### 4.2. Study Selection and Quality

M Aljofan, and A Moldasheva conducted the study search, selection, and quality assessment. Disagreements were solved by discussion with A Zhakupova. The quality of the studies included was assessed according to the quality of the body of evidence in The GRADE approach (see Reference [86]).

### 4.3. Types of Study Selected

Experimental studies include in vitro and/or in vivo studies.

### 4.4. Types of Outcome

The primary measure of interest is the anti-breast cancer mechanism of action of metformin. The secondary measure was all other reported anti-cancer effects of metformin.

### 4.5. Data Extraction

The extracted data included the name of the first author; publication year; methodology; and findings.

### 4.6. Publication Bias and Limitations

This study aimed to analyze the reported anti-breast cancer mechanisms of metformin; therefore, in vitro and relevant in vivo studies were included. Some studies were excluded from the review due to subject duplication, language, and no/limited access to articles. Also, the anti-cancer activity of metformin is relatively a new topic; thus, many reported mechanisms have not yet been reconfirmed.

## 5. Conclusions

This review provides an extensive overview of the antiproliferative effect of metformin on breast cancer cells. In addition to metformin’s known effect in lowering glucose levels, its effect on the induction of apoptosis (including ROS-mediated) and ferroptosis, inhibition of EMT and stem cell-like phenotypes, reduction of cholesterol levels, and its sensitivity to glucose levels provide valuable information on the possible mechanisms of action of metformin treatment in breast cancer cells.

In conclusion, this systematic review confirms the antiproliferative effect of metformin in breast cancer cells. The anti-cancer effect of metformin is not mediated by a single determinant but rather by the alteration of several critical signaling pathways that work together to inhibit cell proliferation, cell migration, angiogenesis, and to induce cell death.

## 6. Future Directions

Although the effect of metformin treatment on cell growth and proliferation was pronounced, these results should be interpreted with caution before treating patients with breast cancer. Large-scale clinical trials on breast cancer patients with and without diabetes would help in understanding the underlying mechanisms of metformin and to evaluate its effect against different sub-types. A possible direction would be to test the effect on postmenopausal women and those with metabolic syndrome, as the effect of metformin treatment may be even more pronounced. Since metformin treatment in breast cancer is a newly developing direction, there are several open questions, including regarding the dosage and duration effect, combination treatment with existing therapies, long-term effects on overall survival, and cancer recurrence, as well as personalized treatment of breast cancer patients based on the genetics and metabolomic profiles of the patients.

## Figures and Tables

**Table 1:** List of studies in the review.

No.	Reference Number	Title	Year	Study Design	Outcome
1	[25]	Metformin induces apoptosis and cell cycle arrest mediated by oxidative stress, AMPK and FOXO3a in MCF-7 breast cancer cells	2014	The study assessed metformin’s ability to inhibit the proliferation of MCF-7 cancer cells as well as its mode of action	Metformin induced cell cycle arrest, apoptosis, oxidative stress, and activation of AMPK and FOXO3a in MCF-7 cell line
2	[26]	Metformin exhibited anti-cancer activity by lowering cellular cholesterol content in breast cancer cells	2019	The study investigated the effect of metformin on cholesterol levels in MDA MB-231 breast cancer cells	Metformin exerted its anti-cancer effects by reducing the amount of cholesterol in breast cancer cells
3	[27]	Metformin induces apoptosis and down-regulates pyruvate kinase M2 in breast cancer cells only when grown in nutrient-poor conditions	2015	The study evaluated how nutrients’ availability changes the metformin anti-cancer effect in MCF7, SKBR3, and MDA-MB-231 cells	In nutrient-deficient conditions, metformin caused apoptosis and PKM2 down-regulation in breast cancer cells
4	[28]	Metformin promotes apoptosis in primary breast cancer cells by down-regulation of cyclin D1 and up-regulation of P53 through an AMPK-alpha independent mechanism	2021	The effect of metformin on expression of AMPK-alpha, cyclin D1, Tp53, and apoptosis was evaluated in primary breast cancer cells	Metformin regulated the transcription of cyclin D1 and p53 in breast tumor cells, preventing cell proliferation and causing apoptosis through an AMPK-alpha-independent mechanism
5	[29]	Metformin induces apoptosis via a mitochondria-mediated pathway in human breast cancer cells in vitro	2016	The study examined the cytotoxic action of metformin in human breast cancer cells MDA-MB-231 and MDA-MB-435	Metformin-induced ROS accumulation leads to the induction of intrinsic-apoptosis
6	[30]	Metformin induces PRODH/POX-dependent apoptosis in breast cancer cells	2022	The study evaluated the effect of metformin on PRODH/POX-dependent apoptosis in wild-type MCF-7 cells (MCF-7WT) and POX knockdown MCF-7 cells (MCF-7crPOX cells)	Metformin induces apoptosis by up-regulating PRODH/POX-dependent ROS formation
7	[31]	Metformin induces degradation of mTOR protein in the breast cancer cells	2016	The study evaluated the mechanisms underlying elevated levels of mTOR in MCF-10A, MCF-7, and MDA-MB-231 breast cancer cell lines	Metformin induced degradation of mTOR by sequestering it in the perinuclear aggressome
8	[32]	Metformin induces ferroptosis by targeting the miR-324-3p/GPX4 axis in breast cancer	2021	The study investigated the effect of metformin on ferroptosis induction and potential underlying mechanism in MDA-MB-231 and MCF-7 and in MB-231 xenografts (in vivo)	Metformin promoted ferroptosis in breast cancer in vitro and in vivo through the regulation of the miR-324-3p/GPX4 axis
9	[33]	Metformin induces ferroptosis by inhibiting UFMylation of SLC7A11 in breast cancer	2021	The effect of metformin on ferroptosis induction and possible underlying mechanisms were evaluated in MCF-7 and T47D cell lines	Metformin induces ferroptosis in breast cancer cells by decreasing UFMylation of SLC7A11
10	[34]	Metformin reverses the mesenchymal phenotype of primary breast cancer cells through STAT3/NF-κB pathways	2019	The ability of metformin to reduce IL-6-induced EMT was evaluated in eight primary breast cancer cells	By keeping STAT3 and NF-kB from activation, metformin prevented the EMT, cell proliferation, and migration of breast cancer cells that were induced by IL-6
11	[35]	Metformin suppresses triple-negative breast cancer stem cells by targeting KLF5 for degradation	2017	The mechanism of action of metformin was studied in triple-negative breast cancer cells (HCC1806 and HCC1937)	Metformin inhibited TNB BREAST CANCER stem cells through the PKA-GSK3-KLF5 signaling pathway
12	[36]	Metformin mediates induction of miR-708 to inhibit self-renewal and chemoresistance of breast cancer stem cells through targeting CD47	2019	The relevance of miR-708 and its downstream target CD47 for the anti-cancer activity of metformin was assessed in MDA-MB-231 and MCF-7 stem cells	Metformin regulated the miR-708/CD47 axis to eradicate breast cancer stem cells and enhance chemosensitivity
13	[37]	The antitumor effect of metformin is mediated bymiR-26a in breast cancer	2016	MDA-MB-231, MDA-MB-468, and MCF-7 breast cancer cell lines were used to test the anti-cancer effect of metformin	Metformin significantly decreased the viability of breast cancer cells by mediating the up-regulation of miR-26a and down-regulation of EHZ2.
14	[38]	Metformin exhibits antiproliferative activity in breast cancer via miR-483-3p/METTL3/m6A/p21 pathway	2021	The study evaluated the effect of metformin on m6A modification by METTL3 in primary breast cancer cells	Metformin inhibited breast cancer cell proliferation via the miR-483-3p/METTL3/m6A/p21 pathway
15	[39]	Metformin inhibits metastatic breast cancer progression and improves chemosensitivity by inducing vessel normalization via PDGF-B down-regulation	2019	The study evaluated the effect of metformin on the remodeling of abnormal vasculature and angiogenesis in MDA-MB-231 and murine 4T1 cell lines	Metformin reduced excessive angiogenesis and enhanced vascular maturity and functionality by lowering tumoral PDGFB
16	[40]	Metformin suppresses the proliferation and invasion of drug-resistant breast cancer cells by activation of the Hippo pathway	2020	The study evaluated the effect of metformin on the Hippo signaling pathway in drug-sensitive (MCF-7) and drug-resistant (LCC2 and MCF7/TAX) cell lines	Metformin activated Hippo pathway by increasing KIBRA and FRMD6 in drug-sensitive MCF-7, whereas in drug-resistant cell lines metformin increased expression of SCRIB leading to inhibition of YAP nuclear localization
17	[41]	High glucose represses the antiproliferative and pro-apoptotic effect of metformin in triple-negative breast cancer cells	2019	The study evaluated the effect of different glucose concentrations on the antiproliferative effect of metformin in MDA-MB-231 and MDA-MB-468 breast cancer cells	Metformin inhibited the mTOR pathway and its downstream components under zero-glucose conditions

## Data Availability

Not applicable.
